# Antithrombotic Therapy Recommendations in the European Society of Cardiology Guidelines: How Robust Are the Randomized Controlled Trials Underpinning Them?

**DOI:** 10.1055/s-0041-1725043

**Published:** 2021-04-14

**Authors:** Catarina M. dos Santos, Luísa Prada, Cláudio David, João Costa, Joaquim J. Ferreira, Fausto J. Pinto, Daniel Caldeira

**Affiliations:** 1 Faculdade de Medicina, Universidade de Lisboa, Lisboa, Portugal; 2 Laboratory of Clinical Pharmacology and Therapeutics, Faculdade de Medicina, Universidade de Lisboa, Lisboa, Portugal; 3 Instituto de Medicina Molecular, Faculdade de Medicina, Universidade de Lisboa, Lisboa, Portugal; 4 Centro de Estudos de Medicina Baseada na Evidência, Faculdade de Medicina, Universidade de Lisboa, Lisboa, Portugal; 5 Serviço de Cardiologia, Hospital Universitário de Santa Maria (CHULN), CAML, Centro Cardiovascular da Universidade de Lisboa—CCUL, Faculdade de Medicina, Universidade de Lisboa, Lisboa, Portugal

**Keywords:** anticoagulant agents, antiplatelet agents, cardiovascular system, fibrinolytic therapy, health planning recommendations

## Abstract

**Introduction** Criticisms have been raised against the sole use of *p* -value in interpreting results from randomized controlled trials (RCTs). Additional tools have been suggested, like the fragility index (FI), a measure of a trial's robustness/fragility, and derivative measures. The FI is the minimum number of patients who would have to be converted from nonevents to events, in the group with the least events, for a result to lose statistical significance.

**Objective** This study aimed to evaluate RCT supporting European Society of Cardiology (ESC) guidelines regarding antithrombotics, using the FI and FI-related measures.

**Methods** FI, fragility quotient (FQ), and FI minus LTF lost to follow-up (FI − LTF) were calculated for the RCT underpinning recommendations regarding antithrombotic therapy from the updated ESC guidelines. LTF was compared with FI. Results were calculated for the total group of studies, as per guideline and as per recommendation type.

**Results** Overall, 61 studies were included. The median FI was 24.5 (interquartile range [IQR]: 9.0–60.0) and median FQ was 0.0035 (IQR: 0.0019–0.0056). Median FI − LTF was 2.0 (IQR: 0.0–38.0). Twenty (32.8%) of the studies had one primary or main safety outcome with LTF exceeding FI. Peripheral arterial disease guideline and chronic coronary syndrome guideline had the lowest (2.5; IQR: 1.8–3.3) and the highest (48.5; IQR: 23.8–73.0) FI, respectively.

**Conclusion** The median FI suggests robustness of clinical trials evaluating antithrombotic drugs cited in the guidelines, but about one-third of them had LTF larger than FI. This emphasizes the need for assessing trials' robustness when constructing guidelines.

## Introduction

Antithrombotic therapy, comprising anticoagulant, antiplatelet, and fibrinolytic drugs, is the current key treatment for some of the major cardiovascular diseases. Decisions regarding the treatment of these conditions are routinely made in accordance to guidelines, which are built based on randomized controlled trials (RCTs), when available. Most often (though not always), these RCTs display statistically significant results, a concept based on *p* -value (the chance of obtaining results at least as extreme provided the null hypothesis is true) of <0.05. For long, the *p* -value has received criticism such as the arbitrariness of its cut-off for significance, the fact that it depends on the selected statistical test and, not less importantly, that its true meaning is often misunderstood due to its complexity. 1 2 Consequently, movements have arisen claiming that other measures, with different information, rather than or alongside the theoretical *p* -value threshold of 0.05, should be reported. 3

The fragility index (FI) is one of those measures. Introduced in 1990 by Feinstein 4 and brought back in 2014 by Walsh et al, 5 it is a tool for assessing a trial's robustness. It can be defined as the minimum number of patients who would have to be converted from nonevents to events, in the group with the least events, for the results to lose their statistical significance. The lower it is, the less robust or more fragile a study is considered. 5 6 7 The FI gave rise to other tools. The fragility quotient (FQ) is the ratio between FI and sample size, allowing the evaluation of a study's fragility in relation to its number of participants. A higher FQ represents more robust outcomes. 6 It is useful to compare robustness between clinical trials of different dimensions, where the sole use of the FI may cause misinterpretations. For example, if both study A and study B have an FI of 10, it might be tempting to think both studies are equally robust. However, if study A has a sample size of 100 and study B of 1,000, FQ for study A is 0.1 whereas FQ for study B is 0.01.

Neither FI nor FQ has strict cut-offs under which they must be analyzed. Instead, they are tools that must be interpreted at light of each study's characteristics. Hence, FI is often compared with the number of patients lost to follow-up (LTF). Having an LTF which exceeds the FI in a certain RCT might be a warning sign for fragility. Therefore, the difference between FI and LTF (FI − LTF) can also be used as a measurement for assessing fragility. 8 The highest this value is, the more robust is the study.

The matter of how robust a study is should be particularly important when it supports guideline recommendations. In this investigation, we propose to assess the robustness of the outcomes of RCT underpinning the recommendations regarding antithrombotic therapy in the most recent versions of the European Society of Cardiology (ESC) guidelines 9 through the FI and related measurements. The ESC guidelines were chosen because of their importance for practicing physicians.

## Materials and Methods

### Identification of Studies

We performed a comprehensive search through the ESC web site section “Guidelines & Scientific Documents” in September 2019. The search was updated in December 2020 according to newly published guidelines. All the latest versions of the guidelines were screened and those mentioning antithrombotic therapy (either antiplatelet, anticoagulant, or fibrinolytic) were selected. We surveyed each of the selected guidelines, to identify every recommendation level of evidence (LOE) A or B (the ones which may be supported by RCT) regarding antithrombotic therapy. Their citations were looked up on PubMed. We performed a primary analysis of titles and abstracts. All RCTs that seemed to fit the inclusion criteria were submitted to a secondary full-text analysis. Inclusion criteria were (1) RCT which assessed antithrombotic therapy in at least one arm; (2) 1:1 random allocation ratio; (3) two parallel arms, two-by-two factorial design or more than two parallel arms, if the recommendation focused only on two of them; (4) at least one dichotomous primary outcome or main safety outcome as statistically significant ( *p* < 0.05 or a 95% confidence interval [CI] that excluded zero, as stated in each trial) for a null hypothesis that no difference existed. Since publicly available data were employed, institutional review board approval was not applicable.

### Data Extraction

First, we retrieved all recommendations on antithrombotic therapy from the ESC guidelines, its LOE and class of recommendation. Then, from each corresponding RCT, data was extracted onto a prepiloted form (Microsoft Excel spreadsheet). Data collection focused on primary and main safety outcomes. It included study identification, control, intervention, population, sample size, control and experimental group sizes, outcome description, number of events in the control and experimental groups, *p* -value, CI, and total LTF.

### Study Outcomes

The primary outcome of this investigation was the fragility/robustness of RCT underpinning the recommendations from ESC guidelines regarding antithrombotic therapy, assessed through the FI, FQ, and FI − LTF.

### Calculating FI, FQ, and FI − LTF

The FI was calculated for each outcome using an online calculator (available at: https://clincalc.com/Stats/FragilityIndex.aspx ) which follows the method described by Walsh et al; adding an event from the group with the smallest number of events and subtracting a nonevent from the same group, so as to keep the total number of participants constant. Then, a two-sided Fisher's exact test is recalculated. The process is automatically repeated by the calculator until the *p* -value is 0.05 or higher. 5 10 FQ was calculated by dividing each FI for the respective sample size. 6 7 FI − LTF was calculated by performing a regular subtraction but, if the result was negative (i.e., if the LTF outweighed the FI), it was considered as zero. 8

### Statistical Analysis

FI, FQ, and LTF median and interquartile ranges (IQRs) were calculated for the whole group of included studies, as per guideline, as per class of recommendation, and as per LOE. To avoid overvaluing studies repeatedly cited in guidelines, we excluded the repetitions under the same guideline topic for the purposes of global analysis and analysis per guideline. LTF was compared with the FI for each outcome. We also calculated FI − LTF for the complete group of studies, as well as its median and IQR. Categorical values were stated as counts and percentages. Spearman's correlation ( *R* ) was used to assess the relationship between FI and sample size, FI and recalculated *p* -value, FI and event rate, and FI − LTF and recalculated *p* -value. *p* -Values for the correlations were calculated through a two-tailed Student's *t* -test. All statistical analysis was done through the Microsoft Excel spreadsheet, apart from calculation of *p* -values. These were calculated through the ClinCalc online calculator, employing Fisher's exact test. 10

## Results

### Selection of Trials and Data Analysis

**Fig. 1:**
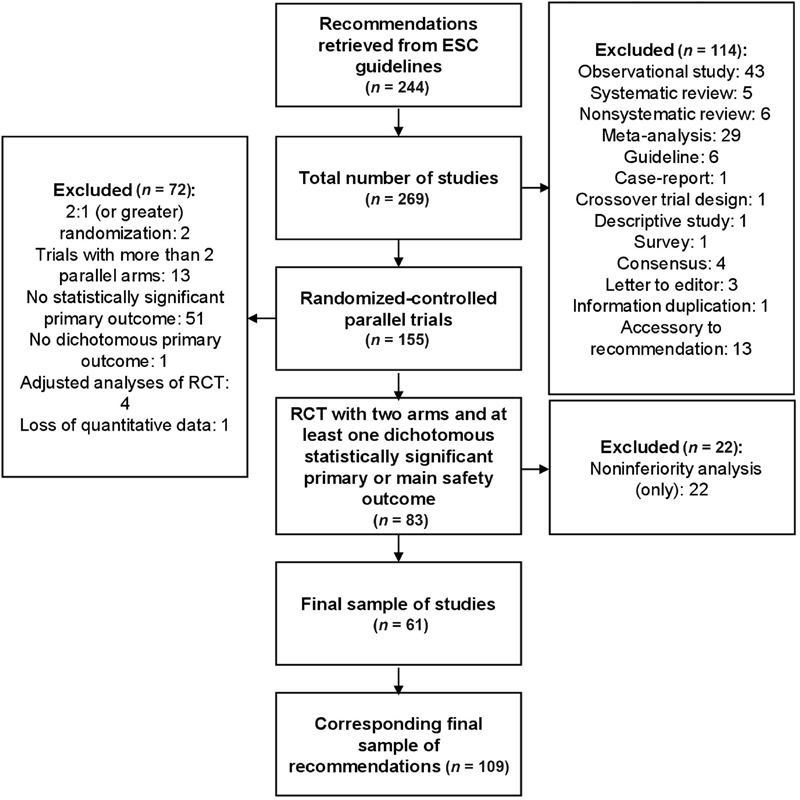
Flow diagram of included and excluded studies. ESC, European Society of Cardiology; RCT, randomized controlled trials.

A total of 18 ESC guidelines 11 12 13 14 15 16 17 18 19 20 21 22 23 24 25 26 27 28 were initially identified as mentioning in any way antithrombotic drugs, with 244 corresponding recommendations. This translated into a sum of 269 studies. One hundred and fifty-five were parallel-arm RCT of which 83 had two arms (or were two-by-two factorial trials or trials with more than two arms of which just two concerned the recommendation under which they were cited) and at least one statistically significant primary or main safety outcome. After excluding 22 trials which presented only noninferiority analyses, we were left with a final sample of 61 studies, with 109 corresponding recommendations from 12 guidelines. 11 12 13 14 15 16 17 18 19 20 26 27 Reasons for exclusion can be found in Fig. 1 .

The median sample size of the studies included was 2,524 (IQR: 855–10,253). The details from the studies and endpoints are presented in Table 1 .

**Table 1:** Characteristics of randomized controlled trials cited in the guidelines

**Characteristics**	***n*** **(%)/median (IQR)**
Number of trials	61
Sample size	2,524.0 (855.0–10,252.8)
Number of control patients	1,270.0 (428.5–5,117.3)
Number of intervention patients	1,254.0 (426.5–5,135.5)
Number of patients LTF	13.0 (3.5–39.5)
Recalculated *p* -value a	62 b (100)
< 0.001	23 (37.1)
0.01–0.001	17 (27.4)
0.05–0.01	18 (29.0)
≥0.05	4 (6.5)

Abbreviations: IQR, interquartile range; LTF, lost to follow-up.

Note: Numbers reported as total (%) or median (IQR).

a *p* -Values calculated using Fisher's exact test.

b One study with factorial two-by-two design was counted twice, since the two pairs of arms were analyzed as individual studies.

There was a total of 109 recommendations in our analysis ( Table 2 ). Most studies analyzed were used to support recommendations class I and LOE A.

**Table 2:** Number of randomized controlled trials supporting different classes of recommendation and levels of evidence

**Total number of recommendations**	**Recommendations = 109**	** RCT = 77 a**
Class I	52	34
Class IIA	27	19
Class IIB	24	18
Class III	6	6
LOE A	63	49
LOE B	46	28
LOE C	0 b	0

Abbreviations: LOE, level of evidence; RCT, randomized controlled trials.

a Number of studies supporting each class/LOE. Studies were counted more than once when they supported recommendations with different class/LOE.

b Since we only included RCT, there are no recommendations LOE C.

### FI, LTF, and FI − LTF

**Fig. 2:**
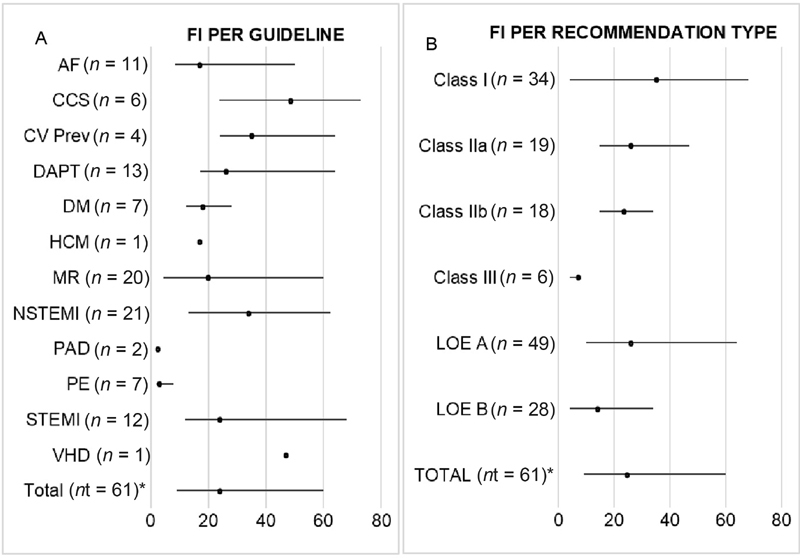
Fragility Index per Guideline and per Type of Recommendation. ( **A** ) Fragility index per guideline. ( **B** ) Fragility index per type of recommendation. Results presented as median and interquartile range. AF, atrial fibrillation; CCS, chronic coronary syndrome; CV Prev, cardiovascular prevention; DAPT, dual antiplatelet therapy; DM, diabetes mellitus; FI, fragility index; HCM, hypertrophic cardiomyopathy; LOE, level of evidence; MR, myocardial revascularization; *n* , number of studies supporting each guideline; NSTEMI, non-ST elevation myocardial infarction; Nt, number of studies supporting all guidelines; PAD, peripheral arterial disease; PE, pulmonary embolism; STEMI, ST elevation myocardial infarction; VHD, valvular heart disease. *Nt differs from the sum of all N because some studies appear in more than one guideline.

The median FI for all 61 trials was 24.5 (IQR: 9.0–60.0). Characteristics of each included study, as well as respective FI, FQ, and FI − LTF can be found in the Supplementary Tables S1 and S2 . The median FI and IQR as per guideline is presented in Fig. 2A . The guideline on peripheral arterial disease 15 had the lowest FI (2.5; IQR: 1.8–3.3). The chronic coronary syndrome guideline 20 had the highest FI (48.5; IQR: 23.8–73.0; Table 3 ).

**Table 3:** FI, FQ, and LTF dispersion as per guideline, class of recommendation, and LOE

**Guideline**	**FI Q1**	**FI Q2**	**FI Q3**	**FQ Q1**	**FQ Q2**	**FQ Q3**	**LTF Q1**	**LTF Q2**	**LTF Q3**
AF ( *n* = 11)	8.5	17.0	50.0	0.0041	0.0082	0.0146	3.0	6.5	7.8
CCS ( *n* = 6)	23.8	48.5	73.0	0.0033	0.0049	0.0287	6.8	27.5	44.0
CV prev ( *n* = 4)	24.0	35.0	64.0	0.0019	0.0035	0.0048	13.0	14.0	255.0
DAPT ( *n* = 13)	17.0	26.0	64.0	0.0019	0.0035	0.0049	13.0	14.0	255.0
DM ( *n* = 7)	12.3	18.0	28.3	0.0013	0.0022	0.0030	14.0	91.5	255.0
HCM ( *n* = 1)	17.0	17.0	17.0	0.0023	0.0023	0.0023	43.0	43.0	43.0
MR ( *n* = 20)	4.5	20.0	60.0	0.0019	0.0039	0.0051	10.0	14.0	80.3
NSTEMI ( *n* = 21)	13.0	34.0	62.5	0.0019	0.0035	0.0050	11.5	16.0	44.0
PAD ( *n* = 2)	1.8	2.5	3.3	0.0154	0.0308	0.0462	10.5	21.0	31.5
PE ( *n* = 7)	2.0	3.0	8.0	0.0030	0.0033	0.0119	5.0	7.0	14.0
STEMI ( *n* = 12)	12.0	24.0	68.0	0.0012	0.0019	0.0044	7.0	14.0	42.0
VHD ( *n* = 1)	47.0	47.0	47.0	0.0835	0.0835	0.0835	2.0	2.0	2.0
Class I ( *n* = 34)	4.0	35.0	68.0	0.0019	0.0041	0.0058	7.0	13.0	16.3
Class IIa ( *n* = 19)	14.8	26.0	47.0	0.0026	0.0035	0.0094	6.0	37.0	255.0
Class IIb ( *n* = 18)	14.8	23.5	34.0	0.0018	0.0026	0.0036	10.0	139.0	255.0
Class III ( *n* = 6)	4.0	7.0	8.0	0.0017	0.0019	0.0030	3.0	5.5	19.3
LOE A ( *n* = 49)	10.0	26.0	64.0	0.0019	0.0039	0.0059	9.0	14.0	255.0
LOE B ( *n* = 28)	4.0	14.0	34.0	0.0018	0.0028	0.0041	6.0	14.0	44.0
Total (Nt = 61) a	9.0	24.5	60.0	0.0019	0.0035	0.0056	7.0	14.0	139.0

Abbreviations: AF, atrial fibrillation; CCS, chronic coronary syndrome; Class, class of recommendation; CV prev, cardiovascular prevention; DAPT, dual antiplatelet therapy; DM, diabetes mellitus; FI, fragility index; FQ, fragility quotient; HCM, hypertrophic cardiomyopathy; LOE, level of evidence; LTF, lost to follow-up; MR, myocardial revascularization; *n* , number of studies supporting each guideline/class/LOE; NSTEMI, non-ST elevation myocardial infarction; Nt, number of studies supporting all guidelines; PAD, peripheral arterial disease; PE, pulmonary embolism; Q1/Q2/Q3, quartile 1/2/3; STEMI, ST elevation myocardial infarction; VHD, valvular heart disease.

a Nt differs from the sum of all *n* because some studies appear in more than one guideline.

Results for FI median and IQR as per class of recommendation and LOE can be found in Fig. 2B . Recommendations class III had the lowest FI (7.0; IQR: 4.0–8.0).

Sixteen (26.2%) of the 61 trials did not disclose number of LTFs. For the totality of studies which did, median LTF was 14.0 (IQR: 7.0–139.0). Median LTF was 13.0 (IQR: 7.0–16.3) for class I; 37.0 (IQR: 6.0–255.0) for class IIa; 139.0 (IQR: 10.0–255.0) for class IIb; and 5.5 (IQR: 3.0–19.3) for class III. LOE A had a median LTF of 14.0 (IQR: 9.0–255.0); LOE B had also a median of 14.0 (IQR: 6.0–44.0). Twenty (32.8%) studies had one primary outcome or main safety outcome in which the LTF exceeded the FI. Four (6.6%) of the 61 trials had a primary outcome or main safety outcome with a FI of 0.

**Fig. 3:**
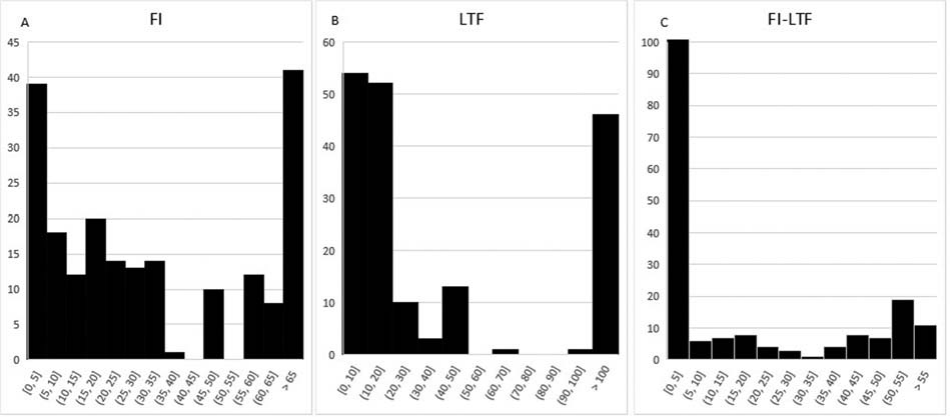
Frequencies of different outcomes. ( **A** ) Frequencies of fragility indices, ( **B** ) patients lost to follow-up and ( **C** ) fragility index minus lost to follow-up. The X-axis represents the FI ( **A** ), the LTF ( **B** ) and the FI − LTF ( **C** ). The Y-axis represents the number of times each value was entered to our global analysis (as described in the section Materials and Methods—Statistical Analysis). FI, fragility index; FI − LTF, FI minus lost to follow-up.

Fig. 3 shows the frequencies for FI, LTF, and FI − LTF. Median FI − LTF was 2.0 (IQR: 0.0–38.0). But 45.0% of the results included had a FI − LTF of 0.

**Fig. 4:**
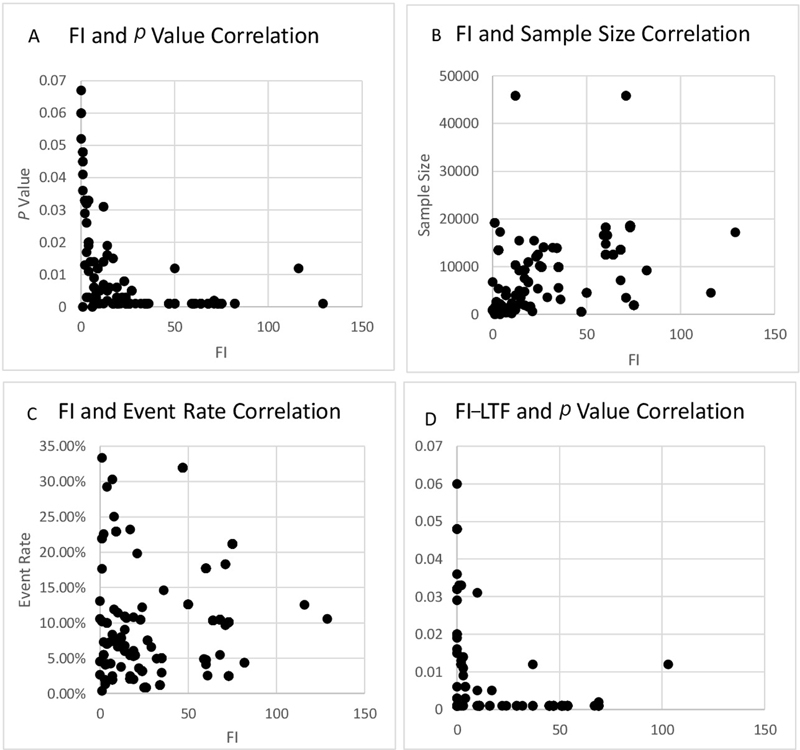
Correlation between fragility index and trial characteristics. ( **A** ) Correlation between fragility index and *p* -value ( *R* =  − 0.77), ( **B** ) fragility index and sample size ( *R* = 0.42), ( **C** ) fragility index and event rate ( *R* = 0.26), and (D) fragility index minus lost to follow-up and *p* -value ( *R* =  − 0.34). FI, fragility index; FI − LTF, FI minus lost to follow-up.

Correlations were *R* =  − 0.77 between FI and *p* -value ( *p* < 0.001), *R* = 0.42 between FI and sample size ( *p* < 0.001), *R* = 0.26 between FI and event rate ( *p* < 0.001), and *R* =  − 0.34 between FI − LTF and *p* -value ( *p* < 0.001; Fig. 4 ).

### Fragility Quotient

Regarding the FQ, its median for the total of studies was 0.0035 (IQR: 0.0019–0.0056). For class I, it is 0.0041 (IQR: 0.0019–0.0058); 0.0035 (IQR: 0.0026–0.0094) for class IIa; 0.0026 (IQR: 0.0018–0.0036) for class IIb; and 0.0019 (IQR: 0.0017–0.0030) for class III. Recommendations LOE A had a FQ median of 0.0039 (IQR: 0.0019–0.0059) and LOE B had 0.0028 (IQR: 0.0018–0.0041). The guideline on valvular heart disease had the highest FQ and the one on myocardial infarction with ST elevation had the lowest FQ.

## Discussion

Our research established the fragility of trial outcomes from 61 RCTs supporting recommendations regarding antithrombotic therapy from the updated versions of ESC guidelines. Our median FI was 24.5 (IQR: 9.0–60.0) which is higher than values reported in previous studies in the cardiovascular field. 29 30

The peripheral artery disease guideline 15 had the lowest FI (2.5; IQR: 1.8–3.3) which suggests the RCTs underpinning it are more fragile. For the analysis of this guideline, only two studies were included, due to restrictions inherent to the FI method. One of the studies included was CAPRIE (clopidogrel versus aspirin in patients at risk of ischaemic events), 31 a trial well known for the fragility of its results, with a borderline statistically significant *p* -value of 0.043 for its main outcome. The other study included by Donaldson et al 32 had a sample size of 65 for its main outcome (and a *p* -value calculated by us of 0.003). Hence, we can here see in practice that both borderline *p* -values and a small sample size contribute to a low FI.

The chronic coronary syndrome guideline, 20 on the other hand, had the highest FI (48.5; IQR: 23.8–73.0). Likely, the high sample sizes of the studies included for this guideline were the determining factor for this high fragility index. Of the six studies, five had more than 1,000 participants and two of these had over 15,000. The smallest sample size in this group was of 563.

The median FI − LTF in our analysis was 2.0 (IQR: 0.0–38.0), meaning that in half of the studies, the number of LTF patients is superior, equal, or very close to the number of patients whose outcome would have to change to render these trials' results nonstatistically significant. The interpretation of these findings, as well as the portion result with an FI − LTF value of 0, is limited by the fact that our analysis included several repetitions, as well as by the fact that it uses total rather than intervention or LTF control. Nonetheless, it may help interpret the overall robustness, if we consider the number of times, a study is cited in the guidelines is directly proportional to its relative importance in their building.

Additionally, in our investigation, 20 of the 61 trials (32.8%) had a primary or main safety outcome in which the LTF outweighed the FI. Conclusions derived from outcomes where the LTF matches or exceeds the FI should be taken with caution. It may have been that those patients vanished from some unfortunate twist of faith (the figurative slip on a banana peel), or that they actually experienced the study's outcome, or both. The comparison of FI and LTF is, therefore, much more valuable than classifying an FI as high or low, a point of difference from the *p* -value. Nevertheless, we must keep in mind, it is unlikely that all patients lost during follow-up would have turned out to be events from the study arm with the lowest event rate. Most likely, they were distributed between the two groups and some of them would end up suffering the study outcome while others would not, had they remained throughout the whole length of the trial. But since we cannot guarantee that this was the case, we have to admit the possibility of the results being changed by the LTF patients, especially in those studies where the LTF largely outweighs the FI.

Another point of interest is outcomes with an FI of 0. We reported a total of four (6.6%) studies with a primary or main safety outcome with an FI of 0, meaning that, without changing the number of events, nonstatistically significant results would have been obtained had the choice of another statistical test. Correspondingly, on Table 1 , we reported four studies with a *p* -value of ≥0.05, determined through Fisher's exact test before calculating the FI. The smallest number of participants reported in this group of RCT was 840. Of the other three studies, one had a sample size of 900 and two had sample sizes over 1,000. Considering none of these numbers is small enough to render Fisher's exact test, the only statistical test suitable, the authors' choice of using other tests in the statistical analysis can be reasonable.

Our analysis also sought to determine the FQ for each outcome. The median FQ was 0.0035 (IQR: 0.0019–0.0056), denoting that there would be no statistical significance if 0.35 in 100 patients had experienced a different event. The guideline on valvular heart disease had the highest FQ (0.0835; IQR: 0.0835–0.0835). For the analysis of this guideline, only one study fit the inclusion criteria. This study, by Dewilde et al, 33 scored an FI of 47, giving this guideline the second highest FI. This, along with a relatively small sample size comparing to other included studies (563 patients allocated) is likely why this guideline had the highest FQ. Both the FI and FQ displayed a decreasing tendency from recommendations from class I to those class III which suggests recommendations in favor of a certain practice are generally supported by more robust trials than those against it. In the case of recommendations, class III, mean evidence for harm (since we only included statistically significant results) in these trials is fragile and there may be instead just a lack of benefit in the intervention.

Similarly to previous findings by Gaudino et al, 30 there was a considerable negative correlation between FI and *p* -value ( *R* =  − 0.77, *p* < 0.001). Also, the FI showed a moderate positive correlation to sample size ( *R* = 0.42, *p* < 0.001) which is in agreement with the values reported by Khan et al ( *R* = 0.32) 29 and Gaudino et al ( *R* = 0.35). 30 The increase in the size of samples seems then to be a candidate for fixing the fragility problem when designing a trial. Although it is true that authors walk a fine line when trying to balance attempts to make a study as robust as possible, while respecting ethical principles which state that a hypothesis should be tested on as few patients as possible, it is also true that fragile studies with unreliable results which do not provide good quality evidence are, themselves, ethically censurable. Furthermore, they call for additional studies on the same topic, requiring, in the end, more participants than it would have, a single more robust trial been performed from the beginning.

## Limitations

Our study has some limitations, the main one being not including systematic reviews with meta-analysis which are a great part of the evidence behind recommendations. Additionally, due to the constraints imposed by the FI method itself, only 61 RCTs were eligible for analysis. Finally, since trials are powered for primary outcomes, we decided to leave out secondary ones. It is also important to emphasize that we selected only trials referred in the guidelines, which means we might be at risk of study selection bias. Even though it is known that guideline recommendations are increasing, LOE A (higher level) and class-I or -III recommendations are decreasing. Therefore, it is important to have tools to critically appraise the evidence in the setting of guidelines. 9

Besides the restrictions which partially shaped our study, others are worthy of note: the FI may not be suitable for time-to-event outcomes, particularly when the number of events in the control and experimental groups is similar, but there is a marked difference in their timings. 5 This is mostly important for trials in the area of oncology; additionally, since it is not a measure of effect (much like the *p* -value) it cannot be used on its own to interpret the result of a trial. 34 Finally, some investigations 35 have shown strong correlation between the FI and *p* -value, leading some authors to state this may be a superfluous tool. 34

## Conclusion

The FI has not come to replace statistical significance. In fact, it is an absolute measure, like others which exist (as the number needed to treat/harm) that aids physicians in better understanding the robustness of trials beyond relative risks and *p* -values. The results of our analysis show that most of the statistically significant studies cited in guidelines to address clinical recommendations have a good fragility index. This means that more than a few additional events are required to cause loss of statistical significance. In our view, the FI, as the most intuitive and thus far studied fragility tool here presented, should be taken into account, when applicable, in the creation of future recommendations for clinical practice guidelines, alongside the *p* -value and CI.
