# Supplementary material for: Antithrombotic Therapy Recommendations in the European Society of Cardiology Guidelines: How Robust Are the Randomized Controlled Trials Underpinning Them?
Source: TH Open. 2021 Apr 14;5(2):e125–33. doi: 10.1055/s-0041-1725043 (PMC8046518; doi:10.1055/s-0041-1725043)
Supplement: Supplementary file 1 — Supplementary Material [file 10-1055-s-0041-1725043-s200065.pdf]

Supplementary Table S1 Characteristics and main results of included studies

Authors	Year	Drug class	Outcome	Sample size analyzed	Number of events	Event rate (%)	LTF	Reported *p*-value	FI	FQ	FI – LTF
Lorenz et al	1984	Antiplatelet	Other vascular events	93	21	22.58	0	0.006	2	0.0215	2
Donaldson et al	1985	Antiplatelet	Other vascular events	65	19	29.23	0	<0.05	4	0.0615	4
ISIS-2 collaborative group	1988	Antiplatelet	Mortality	17,187	1,820	10.59	N/A	N/A	129	0.0075	N/A
EAFT study group	1993	Anticoagulant	NACE	439	110	25.06	N/A	0.001	8	0.0182	N/A
Gent et al	1996	Antiplatelet	MACCE	19,185	1,960	10.22	42	0.043	1	0.0001	0
Schulman et al	1997	Anticoagulant	VTE	227	26	11.45	14	<0.001	2	0.0088	0
Müller et al	2000	Antiplatelet	Other composite outcome	700	49	7.00	1	0.010	0	0.0000	0
Mehta et al	2001	Antiplatelet	MACCE variation	2,658	145	5.46	0	0.030	2	0.0008	2
Taniuchi et al	2001	Antiplatelet	Medication adherence	1,016	27	2.66	N/A	0.043	0	0.0000	N/A
Yusuf et al	2001	Antiplatelet	MACCE	12,562	1,301	10.36	13	<0.001	64	0.0051	51
Steinhubl et al	2002	Antiplatelet	MACCE	2,116	211	9.97	23	N/A	12	0.0057	0
Andersen et al	2003	fibrinolysis	MACCE	1,572	170	10.81	97	<0.001	4	0.0025	0
Lee et al	2003	Anticoagulant	VTE	672	80	11.90	N/A	0.002	3	0.0045	N/A
COMMIT Collabortaive Group	2005	Antiplatelet	MACCE	45,852	4,431	9.66	2	0.002	71	0.0015	69
Sabatine et al	2005	Antiplatelet	MACCE variation	3,491	639	18.30	4	<0.001	71	0.0203	67
Montalescot et al	2006	Antiplatelet	Pharmacodynamic outcome	69	23	33.33	N/A	N/A	4	0.0580	N/A
Stone et al	2006	Antiplatelet, anticoagulant	NACE	9,215	1,004	10.90	30	0.015	14	0.0015	0
Yusuf et al	2006	Anticoagulant	MACCE	12,092	1,262	10.44	33	0.008	23	0.0019	0
Mant et al	2007	Antiplatelet, anticoagulant	NACE variation	973	72	7.40	8	0.003	8	0.0082	0
Wiviott et al	2007	Antiplatelet	MACCE	13,608	1,424	10.46	14	<0.001	68	0.0050	54
Peters et al	2008	Anticoagulant	MACCE	5,436	665	12.23	N/A	N/A	24	0.0044	N/A

 © 2021. The Author(s).

Supplementary Table S1 (*Continued*)

Authors	Year	Drug class	Outcome	Sample size analyzed	Number of events	Event rate (%)	LTF	Reported *p*-value	FI	FQ	FI – LTF
Stone et al	2008	Antiplatelet, anticoagulant	NACE	3,602	384	10.66	28	0.005	19	0.0053	0
Bonnefoy et al	2009	Fibrinolytic	Mortality	840	38	4.52	45	0.040	0	0.0000	0
Connolly et al	2009	Antiplatelet	MACCE	7,554	1,756	23.25	43	0.010	2	0.0003	0
Connolly et al	2009	Anticoagulant	Bleeding	2,390	159	6.65	N/A	N/A	10	0.0042	N/A
Wallentin et al	2009	Antiplatelet	MACCE	18,624	1,878	10.08	4185	<0.001	73	0.0039	0
Mehta et al	2010	Antiplatelet	MACCE	17,263	722	4.18	3	0.039	15	0.0009	12
Connolly et al	2011	Antiplatelet, anticoagulant	Stroke	5,599	164	2.93	N/A	<0.001	35	0.0063	N/A
Becattini et al	2012	Antiplatelet	VTE	402	71	17.66	7	0.020	15	0.0373	8
Diener et al	2012	Antiplatelet, anticoagulant	Stroke	4,832	121	2.50	N/A	N/A	17	0.0035	N/A
Easton et al	2012	Anticoagulant	Bleeding	14,765	606	4.10	N/A	N/A	60	0.0041	N/A
Mega et al	2012	Anticoagulant	MACCE	10,350	689	6.66	4280	0.007	12	0.0012	0
Valgimigli et al	2012	Antiplatelet	Bleeding	1,970	107	5.43	7	0.000	0	0.0000	0
Bhatt et al	2013	Antiplatelet	MACCE variation	10,942	579	5.29	2	0.005	19	0.0017	17
Collet et al	2013	Anticoagulant	NACE variation	795	241	30.31	6	0.012	7	0.0088	1
Dewilde et al	2013	Antiplatelet, anticoagulant	Bleeding	563	180	31.97	2	<0·0001	47	0.0835	45
Montalescot et al	2013	Antiplatelet	Bleeding	4,033	79	1.96	3	0.006	7	0.0017	4
Steg et al	2013	Antiplatelet, anticoagulant	Bleeding	2,198	149	6.78	N/A	0.001	20	0.0091	N/A
Mauri et al	2014	Antiplatelet	Other vascular events	9,961	84	0.84	255	<0.001	26	0.0026	0
Meyer et al	2014	Anticoagulant, fibrinolysis	Other composite outcome	1,005	41	4.08	N/A	0.020	1	0.0010	N/A
Napolitano et al	2014	Anticoagulant	VTE	347	52	14.99	101	N/A	10	0.0288	0
Shahzad et al	2014	Anticoagulant	MACCE variation	1,812	131	7.23	2	0.010	0	0.0000	0
Andreozzi et al	2015	Anticoagulant	VTE	615	45	7.32	5	0.025	5	0.0081	0
Bonaca et al	2015	Antiplatelet	MACCE	14,112	1,065	7.55	10	0.004	27	0.0019	17

(*Continued*)

 © 2021. The Author(s).

Supplementary Table S1 (*Continued*)

Authors	Year	Drug class	Outcome	Sample size analyzed	Number of events	Event rate (%)	LTF	Reported *p*-value	FI	FQ	FI – LTF
Couturaud et al	2015	Anticoagulant	Other composite outcome	371	31	8.36	4	0.001	14	0.0377	10
Lee et al	2015	Anticoagulant	Bleeding	900	118	13.11	29	0.004	4	0.0044	0
Franzone et al	2016	Antiplatelet	Bleeding	1,724	93	5.39	N/A	<0.001	14	0.0081	N/A
Rost et al	2016	Anticoagulant	Bleeding	14,024	696	4.96	0	0.002	32	0.0023	32
Anand et al	2017	Antiplatelet, anticoagulant	MACCE	4,996	300	6.00	4	0.005	1	0.0002	0
Calkins et al	2017	Anticoagulant	Bleeding	635	27	4.25	N/A	<0.001	6	0.0094	N/A
Cannon et al	2017	Antiplatelet, anticoagulant	Bleeding	1,962	415	21.15	6	<0.001	75	0.0382	69
Connolly et al	2017	Antiplatelet, anticoagulant	MACCE	16,574	807	4.87	18	<0.0001	59	0.0036	41
Cuisset et al	2017	Antiplatelet	NACE	645	128	19.84	11	<0.01	21	0.0326	10
Eikelboom et al[a]	2017	Antiplatelet, anticoagulant	MACCE	18,278	875	4.79	44	<0.001	60	0.0033	16
Eikelboom et al[a]	2017	Anticoagulant	Bleeding	4,549	573	12.60	13	<0.001	50	0.0110	37
Bowman et al	2018	Antiplatelet	MACCE	15,480	1,401	9.05	139	0.010	7	0.0005	0
Ezekowitz et al	2018	Anticoagulant	Stroke	1,500	6	0.40	1	0.015	1	0.0007	0
Lopes et al	2019	Antiplatelet	Bleeding	4,556	571	12.53	13	<0.001	116	0.0255	103
Mehran et al	2019	Antiplatelet	Bleeding	7,119	391	5.49	68	<0.001	68	0.0096	0
Schupke et al	2019	Antiplatelet	MACCE	4,018	321	7.99	37	0.006	12	0.0030	0
Windecker et al	2019	Anticoagulant	Bleeding	1,092	92	8.20	N/A	<0.001	14	0.0128	N/A
Yasuda et al	2019	Antiplatelet, anticoagulant	Bleeding	2,215	93	4.20	44	0.010	4	0.0018	0

Abbreviations: FI, fragility index; FQ, fragility quotient; LTF, lost to follow-up; MACCE, major adverse cardiovascular and/or cerebrovascular event; N/A, not available; NACE, net adverse clinical events; VTE, venous thromboembolism; ISIS-2, second international study of infarct survival; EAFT, European atrial fibrillation trial; COMMIT, ClOpidogrel and Metoprolol in Myocardial Infarction Trial.
Note: In this table only the main primary outcome taken from each study is represented. In the analysis, additional primary outcomes were used provided they fulfilled the inclusion criteria.
[a]This factorial two-by-two study was counted twice, since the two pairs of arms were analyzed as individual studies.

 © 2021. The Author(s).

Supplementary Table S2 Previous fragility index analyses in the cardiovascular field

Reference	Author	Included studies	Sample size median	Sample size IQR	FI median	FI IQR
29	Khan et al	123	2,466	(1,005–7,513)	13	(5–26)
30	Gaudino et al	160	1,192	(379–2,672)	8	(3–15)
	Current study	57	2,428	(795–10,463)	24	(9–60)
		FQ median	FQ IQR	LTF > FI (%)		
29	Khan et al	0.0042	0.0020–0.0110	30		
30	Gaudino et al	–	–	43		
	Current study	0.0035	0.0018–0.0051	37		

Abbreviations: FI, fragility index; FQ, fragility quotient; IQR, interquartile range; LTF, lost to follow-up; RCT, randomized controlled trials.

 © 2021. The Author(s).
